# Augmented pedicle trajectory applied on the osteoporotic spine with lumbar degenerative disease: mid-term outcome

**DOI:** 10.1186/s13018-019-1213-y

**Published:** 2019-06-06

**Authors:** Guo-ye Mo, Hui-zhi Guo, Dan-qing Guo, Yong-chao Tang, Yong-xian Li, Kai Yuan, Pei-jie Luo, Ten-peng Zhou, Shun-cong Zhang, De Liang

**Affiliations:** 1 First School of Clinical Medicine, Guangzhou University of Chinese Medicine, 12 Airport Road, Baiyun District, Guangzhou, 510405 Guangdong People’s Republic of China; 2 The First Affiliated Hospital of Guangzhou University of Chinese Medicine, Guangzhou, 510407 China

**Keywords:** Lumbar degenerative diseases, Osteoporosis, Cement-augmented, Pedicle screw

## Abstract

**Purpose:**

To compare the safety and efficiency of cement-augmented pedicle screw with traditional pedicle screw technique applied on the patients in the osteoporotic spine with lumbar degenerative diseases.

**Methods:**

Fifty-six patients followed up at least 2 years were enrolled in our institute with retrospectively reviewed from January 2009 to June 2014, diagnosed as lumbar spondylolisthesis, or lumbar stenosis, with *T* score ≤− 2.5 SD of BMD, and received less than three-segment PLIF or TLIF. All patients were divided into 2 groups: 28 (2 males, 26 females) in polymethylmethacrylate-augmented pedicle screw group (PSA) group, the other 28 (3 males, 25 females) in traditional pedicle screw group (TPS). Surgical data including the operation time, intra-operative blood loss, hospitalization day and surgical complications were recorded, as well as the radiological parameters measured from the postoperative X-rays and CT scans containing the rates of fusion, screw loosening, and cage subsidence incidence. In addition, the visual analog scores (VAS) and Oswestry Disability Index (ODI) were evaluated preoperatively and postoperatively.

**Results:**

The average follow-up period was 34.32 months (ranging from 24 months to 51 months). Compared with PSA group, operation time and average hospital stay in the TPS group decreased significantly (*P* < 0.05). While no statistical difference for blood loss between 2 groups (*P* > 0.05). At 2 years postoperation, from CT-scans, 2/172 screws loosening and 1/56 segment non-union occurred in PSA group, with significantly lower incidence than those in TPS group (8/152 screws loosening and 6/50 segments non-union occurred, *P* < 0.05). Regarding the cage subsidence, 24 segments found height loss (5.30 ± 1.92 mm) in PSA group without difference compared with that of 19 segments (4.78 ± 1.37 mm) in TPS group (*P* > 0.05). Besides, the number and the location of cages and the leakage of the cement were found out little related with the subsidence in the PSA group (*P* > 0.05). After surgeries, VAS and ODI at 1 month, 6 months, 12 months, and last follow-up improved significantly in two groups (*P* < 0.05). There were no significant differences in VAS and ODI preoperatively and postoperatively between 2 groups (*P* > 0.05). In addition, eight patients with asymptomatic trajectory PMMA leakages were detected.

**Conclusion:**

Cement-augmented pedicle screw technique is effective and safe in the osteoporotic spine with lumbar degenerative diseases, with better fusion rates and less screw loosening incidence. There is no difference in the fusion rate and loosening rate between the two groups in the single segment patients; however, there are better fusion rate and lower pedicle screw loosening rate of the PSA group in the double or multiple group patients.

## Introduction

As the aging population accelerates, spates of elder patients are afflicted by the back pain and disability resulted from increasing lumbar degenerative diseases which lead to instability of spine and compression of neural elements. Decompression and fusion surgery combined with pedicle screw fixation system is known as an alternative to address these spinal problems, improving stability and fusion rate [1]. However, fixation failure occurs frequently on these elder patients due to poor bone quality [2]. Many innovations have been developed to increase pullout strength of screws in the osteoporotic spine, such as expanding the length and diameter of the screw, modifying the trajectory, and using an expandable screw and cement-augmented pedicle screw [3–7]. Biomechanical studies have demonstrated increased resistance to failure of the screw-bone interface after augmentation with bone cement. Clinical studies have reported good functional outcomes and very low revision rates with polymethylmethacrylate (PMMA)-augmented screws [8].

However, several issues still remained to be unknown: (1) whether polymethylmethacrylate cement impairs the vertebral blood flow, decreasing bone-graft fusion rates; (2) whether the cement compresses the endplate resulting in increasing cage subsidence risks. We used this technique in our institute in cases of 28 patients with degenerative lumbar diseases and followed up the required stability and the severity of complications.

## Materials and methods

### Study population

All patients provided their informed consent for surgery. The study was approved by the local ethical committee and performed in accordance with the ethical standards of the 1964 Declaration of Helsinki as revised in 2000. From January 2009 to June 2014, surgical procedures were performed by means of a posterior approach using pedicle screws in the lumbar spine for the treatment of lumbar degenerative diseases. Routine lumbar radiographs along with computed tomography (CT) and magnetic resonance imaging (MRI) scans were conducted to confirm the exact nature and level of pathology. Fifty-six patients were furtherly retrospectively reviewed. They were divided into 2 groups; 28 (2 males, 26 females) in polymethylmethacrylate-augmented pedicle screw group (PSA), the other 28 (3 males, 25 females) in traditional pedicle screw group (TPS). All patients have received analgesic drug and physiotherapy treatment for more than 3 months. The patients of PSA group were used bone cement-injectable cannulated pedicle screw fixation. Indication for the use of cemented screws was confirmed by evaluating the degree of osteoporosis in all patients. T score≤− 2.5SD was an indication for this technique [9]. Baseline characteristics for clinical information including age, gender, BMI, BMD, diagnosis, and follow-up were collected. All in both groups take vitamin D and calcium regularly to treat osteoporosis.

### Inclusion and exclusion criteria

Inclusion criteria: (1) The *T* score of bone mineral density (lumbar vertebrae and one femoral neck, measured by dual-energy X-ray absorptiometry (4500-type, Hologic, USA)) is ≤− 2.5SD; (2) the diagnosis was lumbar spinal stenosis or lumbar spondylolisthesis; (3) less than three-segment posterior lumbar interbody fusion(PLIF)/transforaminal lumbar interbody fusion (TLIF); (4) follow-up time ≥ 2 years. Exclusion criteria were as follows: (1) spinal infection or tumor; (2) vertebral fracture in fused segment; (3) degenerative scoliosis; (4) parathyroid glands hyperfunction, ankylosing spondylitis, or osteomalacia.

### Operative methods

Under general anesthesia, with the patient in the prone position, the target segments were approached by a posterior midline incision and the facet joint gradually exposed. In the PSA group, fenestrated pedicle screws (REACH Medical, Shanghai, P.R. China) were inserted into the lumbar vertebra then approximate 1.5 ml bone cement was injected under the monitoring of fluoroscopy. In the TPS group, the pedicle screws were inserted into the lumbar vertebra. Next, the PLIF/TLIF was carried out. A polyether ether ketone (PEEK) interbody cage, autogenous, and allogeneic bones were used.

### Observational parameters

**Fig. 1:**
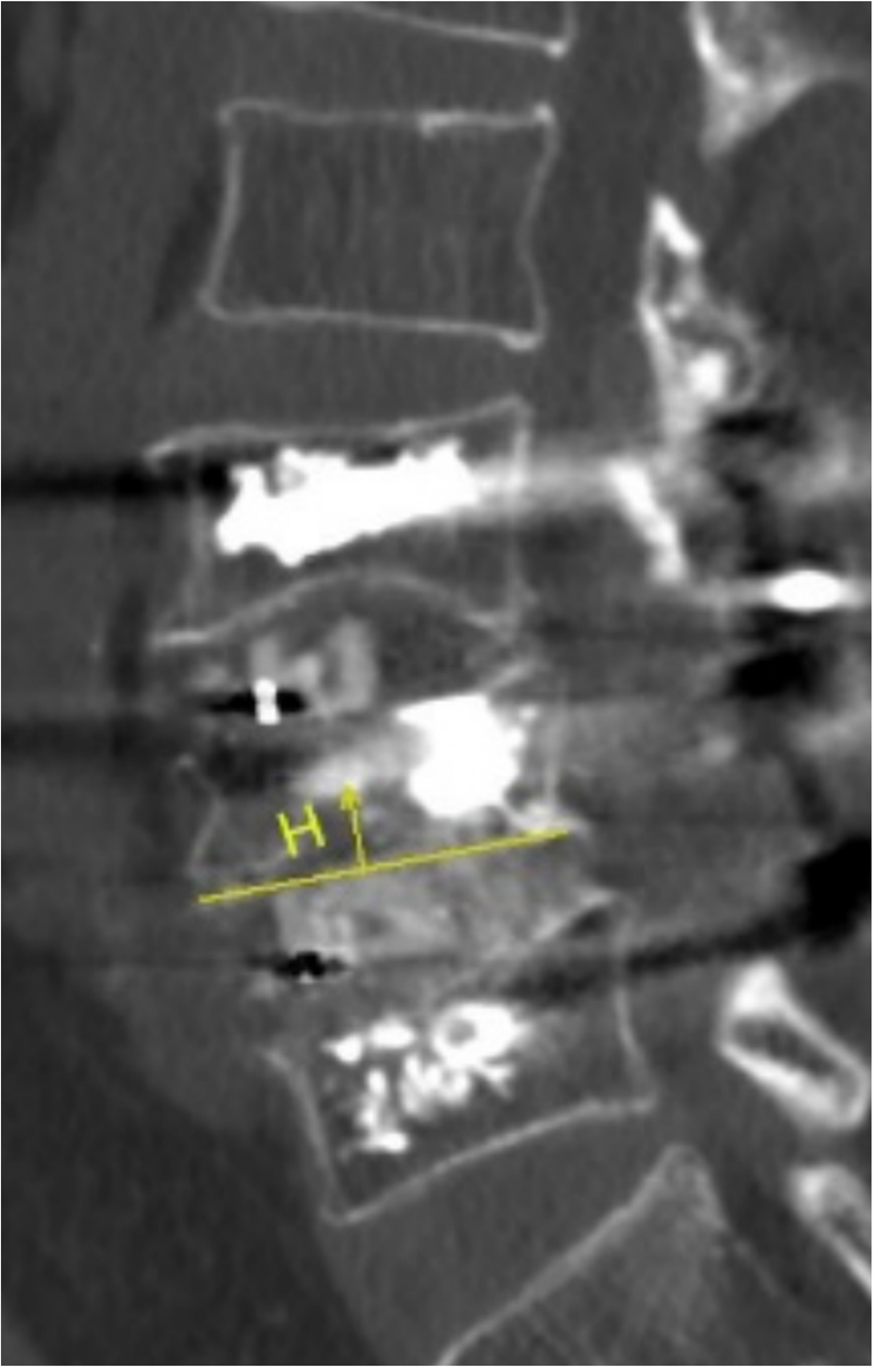
The measuring method of cage subsidence

Surgical data including the operation time, intra-operative blood loss, hospitalization day, and surgical complications were recorded. Radiological parameters measured from the postoperative X-rays and CT scans containing the rates of fusion, screw loosening, lumbar lordosis, and cage subsidence incidence. Radiographic cage subsidence was measured in CT scans from the vertebral endplate to the caudal or cranial margin of the cage (in millimeters) (Fig. 1). The position of the sunk cage was located at anterior, middle, or posterior part of the intervertebral space from the lateral X-ray, and the number was counted from CT. Graft fusion grade was determined with the Bridwell et al. [10] fusion system, only grades 1 and 2 defined as satisfactory fusion. Screw loosening was detected with the translucent shadow around the screw from CT. The data were measured by two experienced spine surgeons. All patients received oral anti-osteoporotic medicine (calcium and vitamin D supplement) at the time being admitted. In addition, the visual analog scores (VAS) and Oswestry Disability Index (ODI) were evaluated preoperatively, at 6 months, 2 years, and the final follow up.

### Statistical analysis

Data collection and statistical analysis were performed using SPPS 22.0 version. Data are reported as the mean, the standard deviation (SD), and the range if continuous, and as the absolute and relative frequencies if categorical. Pre- and postoperative VAS scale scores and ODI values were compared using the *t* test for independent sample and ANOVA analysis for paired sample. The independent-sample t-test was performed to compare demographics. The number and position of the cage, bone cement in the vertebral body, fusion rate and pedicle screw loosening rate were compared by chi-square test. Statistical significance is at a level of significance of 0.05.

## Results

### Results of demographics

There are 28 patients in each group. The results of the demographics of the two group were not statistically significant (Table 1).

**Table 1:** Results of demographics

	PSA group	TPS group	*P* value
Cases	28	28	
Male/female	2/26	3/25	1.000
Diagnosis (LSS/spondylolisthesis)	15/13	18/10	0.587
Age (years)	67.12 ± 1.31	66.04 ± 1.08	0.527
Follow-up time (month)	35.04 ± 7.39	33.61 ± 5.36	0.412
BMD	− 3.02 ± 0.17	− 3.01 ± 0.12	0.971
BMI	24.38 ± 0.72	24.59 ± 0.61	0.828
Size of subsidence cage			
Height (mm)	10.92 ± 0.77	10.96 ± 0.76	0.813
Length (mm)	23.67 ± 2.00	24.37 ± 2.00	0.173
Bone graft			0.752
Allograft bone	7	6	
Autogenous iliac crest bone	21	22	
Fusion segment (single/double/multiple)	6/16/6	12/10/6	0.184

### Results of radiographic evaluation

**Fig. 2:**
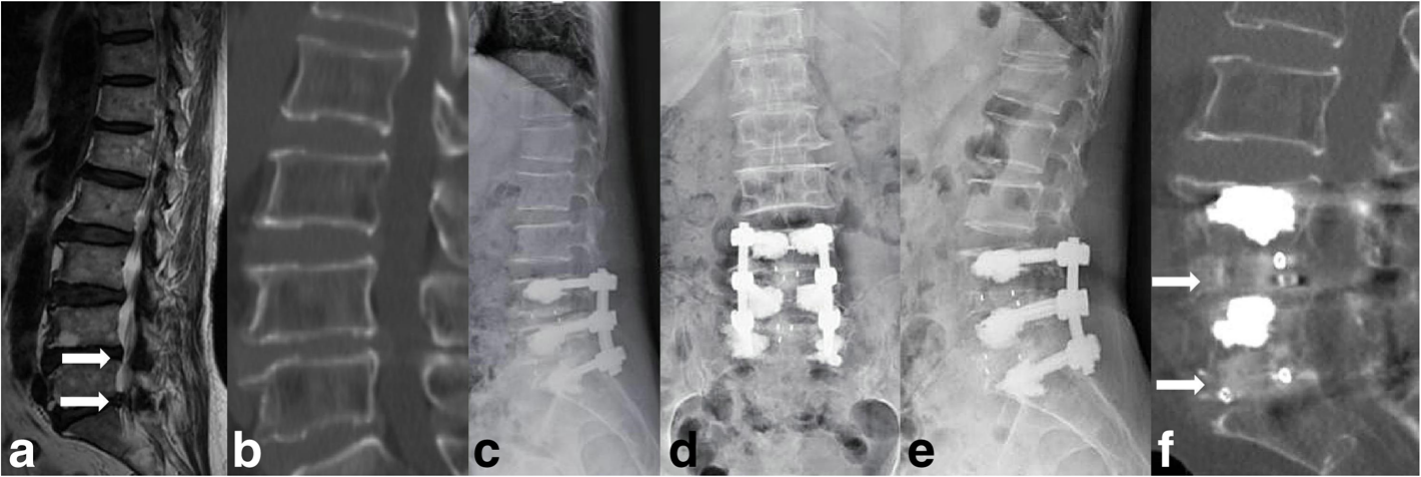
Radiological images of a representative case with polymethylmethacrylate-augmented pedicle screw treatment. **a**–**f** A 67-year-old female with lumbar spinal stenosis in L4/5 and L5/S1 segments. **a** Preoperative MRI image showed obvious spinal stenosis in L4/5 and L5/S1 segments (white arrow). **b** Preoperative CT scanning showed no osteophyte in posterior of vertebrae. **c**–**d** Postoperative X-ray showed the normal position of pedicle screws and the cement in the vertebrae. **e** Lateral X-ray at 36 months after fusion surgery showed normal position of pedicle screws. **f** Lumber CT scanning at 36 months after fusion surgery showed series cancellous bone through L4/5 and L5/S1 intervertebral space (white arrow)

There were two pedicle screws loosening in the PSA and eight pedicle screws loosening in the TPS. The screw loosening rate of PSA group was 1.16%, which was lower than 5.26% of the TPS group. There was no non-union in the single segment of the PSA group, but one non-union segment in the TPS group. However, there was one non-union segment and six non-union segments in double or multiple segments patients of PSA group and TPS group, respectively (Table 2). The fusion rate or PSA group was 98.21%, which higher than 86.00% of TPS group. They were statistically significant. The average operative time, 266.30 ± 14.51 min, and hospital stays, 23.69 ± 1.44 days, of PSA group were greater than TPS group, and they were statistically significant. There were 24 cages subsidence in the PSA group and 19 cages subsidence in the TPS group, and the subsidence height of the PSA group was 5.30 ± 1.92 mm higher than 4.78 ± 1.37 mm of the TPS group. But they were not statistically significant. In the PSA group, 24 fusion segment cages arose subsidence and 32 fusion segment cages were normal. 33.33% cage subsidence associated with cement leakage and the leakage rate of the normal fusion segment cages was 25%, but there was no significant difference. The typical case of PSA group was in Fig. 2. All the cement leakage was paravertebral leakage, without obvious clinical symptoms. The number and position of cage and the leakage of bone cement were not associated with cage subsidence (Tables 3 and 4).

**Table 2:** Details about different fusion segment

	PSA group		TPS group		*P* value
	Non-union segment	Total segments	Non-union segment	Total segments	
Single segment	0	6	1	12	0.485
Double/multiple segment	1	50	6	38	0.030

Note: *P<0.05

**Table 3:** Radiographic evaluation operation result

	PSA group	TPS group	*P* value
Pedicle screws	168	156	
Fusion segments	56	50	
Non-union rate	1/56	7/50	0.028*
Loosening rate	2/168	8/156	0.047*
Lumber lordosis (°)			
Pre-operation	32.71 ± 11.71	29.83 ± 13.87	0.415
Post-operation	32.02 ± 10.41	28.60 ± 11.17	0.251
Final follow-up	34.59 ± 11.80	30.47 ± 10.05	0.173
Operation time (min)	266.30 ± 14.51	204.90 ± 10.35	0.002*
Blood loss (ml)	605.0 ± 121.90	648.10 ± 89.77	0.725
Hospital stays (day)	23.69 ± 1.44	19.44 ± 1.18	0.026*
Number of subsidence cage (one/two)	12/12	11/8	0.606
Position of subsidence cage			0.983
Anterior middle posterior	16	13	
Anterior middle	6	4	
Middle posterior	2	2	
Subsidence height(mm)	5.30 ± 1.92	4.78 ± 1.37	0.606

Note: *P<0.05

**Table 4:** Details about cage subsidence in the PSA group

	Subsidence	Normal	*P* value
Fusion segments	24	32	
Number of cage (one/two)	12/12	17/15	0.817
Position of cage			0.653
Anterior middle posterior	16	20	
Anterior middle	6	8	
Middle posterior	2	4	
Cement leakage	8	8	0.495

Note: *, *P*<0.05

### Clinical results

**Fig. 3:**
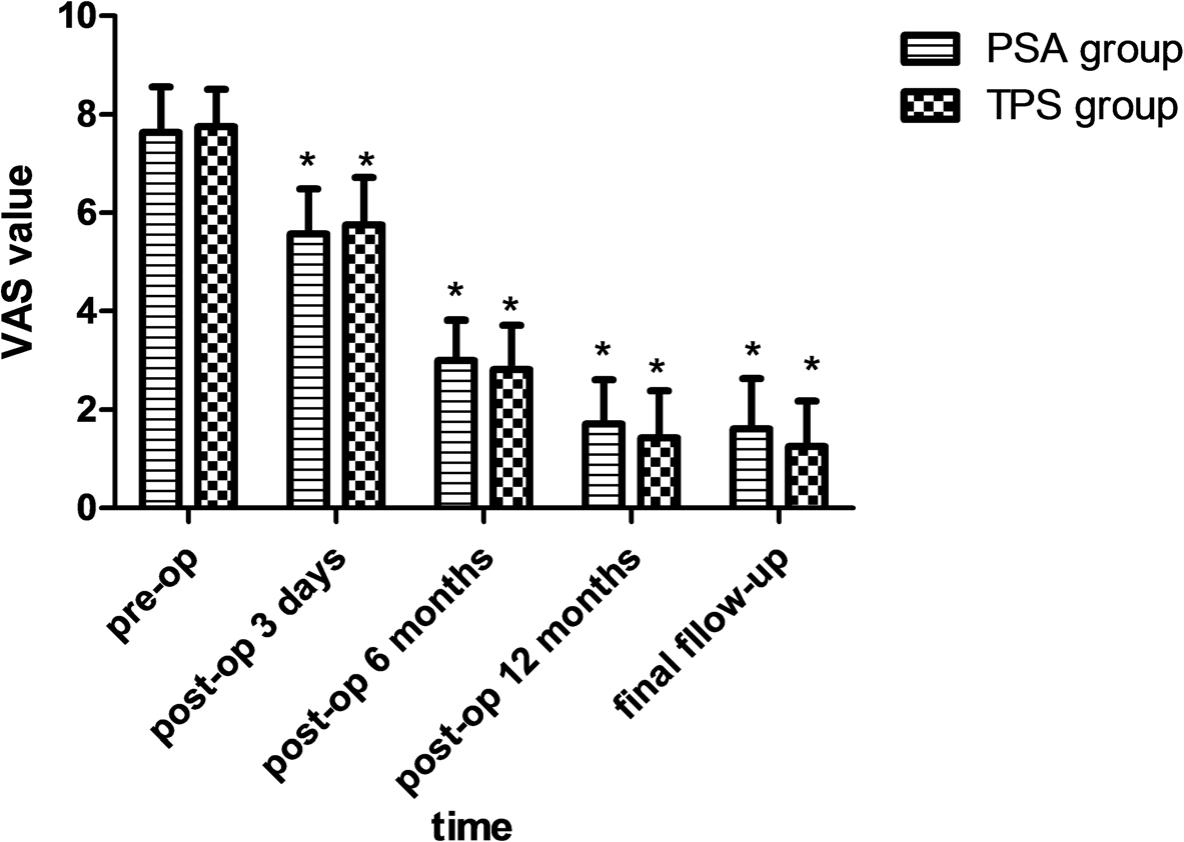
VAS value of the two groups. Note: *, *P*<0.05

**Fig. 4:**
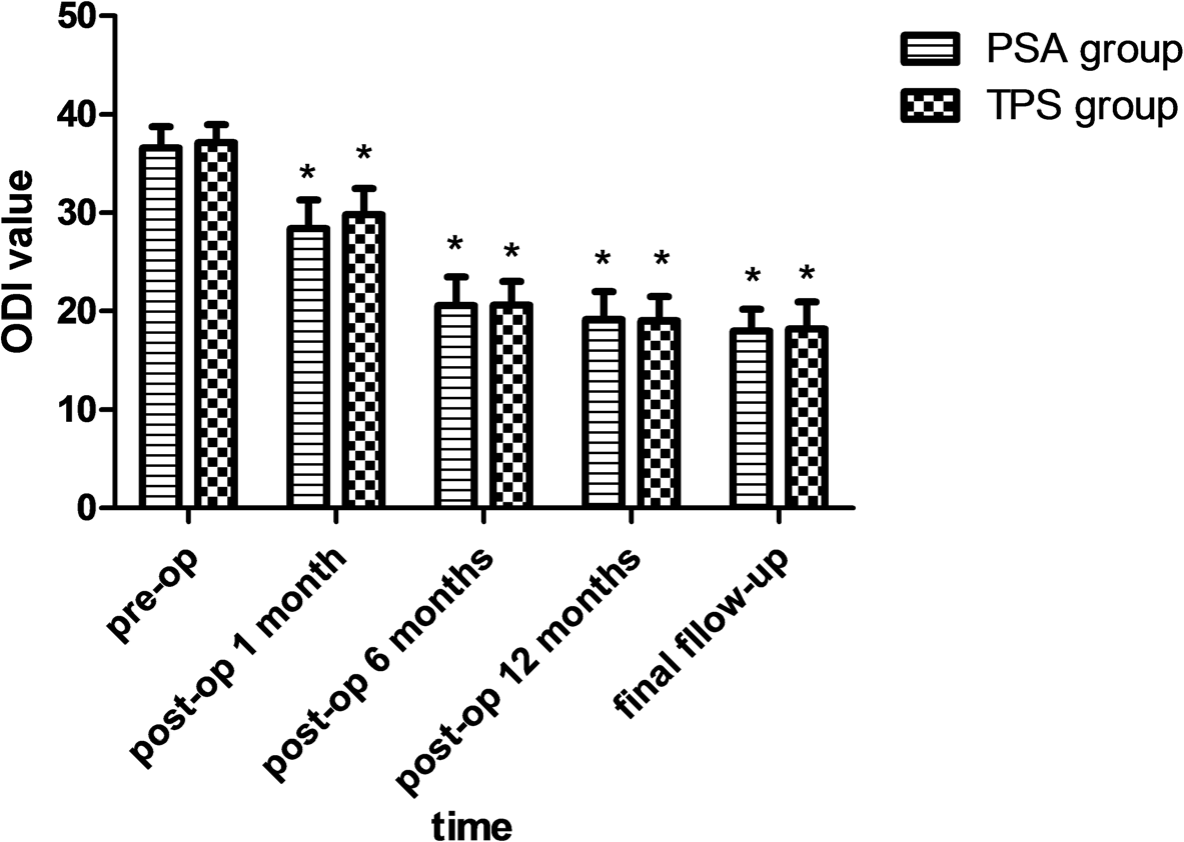
ODI value of the two groups. Note: *, *P*<0.05

The preoperative VAS and ODI of the PSA group were 7.75 ± 0.75 and 36.61 ± 2.17, respectively, and they were higher than the postoperative. Similarly, the preoperative VAS and ODI of TPS group were 7.64 ± 0.91 and 37.14 ± 2.17, respectively, which were higher than the postoperative. They were statistically significant. There was no significant difference between the two groups (Figs. 3 and 4).

## Discussion

One of the goals for treating lumbar degenerative disease is to decompress and achieve the stability of the spine [11]. Pedicle screw loosening reported as the common complication may lead to the failure of fusion in osteoporotic spine with a rate from 0.6 to 11% [5, 12–14]. According to the report, 60% of pull-out strength of pedicle screw comes from the vertebral cancellous bone [15]. The high risk of screw loosening in osteoporotic spine is due to the fragile bone with a rate of 4.1–12.9% [16]. Considering the traditional pedicle screw unsatisfactory performance in the poor-quality spine, cemented-augmented pedicle screws have been an alternative to strengthen the anti-pullout capability by dosing cement carefully through the screws into the vertebral body. Some researchers reviewed cases using 158 cement-augmented pedicle screws, no screw loosening found [16]. In our study, the 178 cement-augmented pedicle screws with a loosening rate of 1.16%, significantly lower than the traditional pedicle screws with a rate of 5.26% in osteoporotic spine at 2-year follow-up. Among the screws loosened, screws at S1 were observed more, probably ascribing to the great shear force based on the aslant anatomic structure of S1.

Successful intervertebral graft bone fusion is based on segmental stability. Screw loosening is reportedly associated with graft bone non-union, pseudoarthrosis, and secondary kyphosis [17, 18]. Pedicle screw augmentation is demonstrated capable to improve the stability of the instrumentation and achieve satisfactory fusion with a rate from 92.50 to 100% in poor spinal bone [19–22]. Similarly, in this study, the fusion rate of the PSA group achieved 98.2%, higher than 86.0% of TPS group. In the PSA group, one case has non-union with pedicle screw loosening, whereas seven cases of TPS group have non-union, including three cases with pedicle screw loosening. The L5/S1 segment non-union case of PSA group is a 56-year-old female suffering from rheumatoid arthritis and long-term use of glucocorticoid. Her *T* value of lumbar bone mineral density is − 3.5, and 2 screws of S1 become loose. The study suggested that there no difference in single segment fusion rate between the two groups. But the non-union rate of PSA group was lower than TPS group in the double or multiple segments.

The goals for inserting cage in intervertebral body are the restoration of intervertebral height, maintainment of spinal balance, and segmental stability. Cage subsidence as a common postoperative complication may result in recollapse of intervertebral foramen, leading to new neurological compression [23]. The rate of cage subsidence was as high as 22% in posterior lumbar interbody fusion [24, 25]. However, we detected 24 segments with cage subsidence (42.86%) in the PSA group and 19 (38%) in TPS group. Because the bone mineral density of patients in this study is lower, the cage subsidence rate of the two groups is higher. The cage subsidence height, (5.30 ± 1.92) mm, in the PSA group is larger than the TPS group, (4.78±1.37) mm, but the difference is not statistically significant. The different position of the cage was revealed to be associated with the difference of lumbar lordotic restoration [11]; however, the paucity of literature reported the association with subsidence and the cage position. The different positions of cage lead to different stress of the vertebral endplate. But based on our result, the position of the cage is not associated with subsidence. We also found that the distribution of bone cement in vertebral body and cement leakage are not related to cage subsidence in the PSA group.

Regarding pain relief and function improvement, PSA group enjoyed a satisfactory result as much as TPS group did. The operation time and hospital stay of PSA group are longer than TPS group. Using polymethylmethacrylate-augmented pedicle screw needs more operation time in the PSA group with more blood loss than TPS group, although there is no statistical significance.

There some limitations to the study. Firstly, it is a retrospective analysis with small sample size. A prospective study is necessary to further confirm the differences observed. Theoretically, the cage subsidence should subtract in PSA group with less pressure from endplate on the cage due to the strength bared more by screws augmented providing enough segmental stability than traditional screw. But our statistic result is negative. We need further enlarged sample to confirm it. Thirdly, we blended the cases with different numbers of fusion segments to analyze, as a consequence, which may increase the bias of conclusion because the bone fusion, screw loosening, and cage subsidence were affected by pressure differed in single, double, or multiple segmental fixations.

## Conclusion

Cement-augmented pedicle screw technique is effective and safe in the osteoporotic spine with lumbar degenerative diseases, with better fusion rates and less pedicle screw loosening incidence. There is no difference in the fusion rate and pedicle screw loosening rate between the two groups in the single segment patients; however, there are better fusion rate and lower pedicle screw loosening rate of the PSA group in the double or multiple group patients.

## Data Availability

The datasets used and analyzed during the current study are available from the corresponding author on reasonable request.

## Abbreviations

CT: Computed tomography
MRI: Magnetic resonance imaging
ODI: Oswestry Disability Index
PLIF: Posterior lumbar interbody fusion
PMMA: Polymethylmethacrylate
PSA: Polymethylmethacrylate-augmented pedicle screw group
SD: Standard deviation
TLIF: Transforaminal lumbar interbody fusion
TPS: Traditional pedicle screw
VAS: Visual analog scores
